# An Efficient Recommendation Filter Model on Smart Home Big Data Analytics for Enhanced Living Environments

**DOI:** 10.3390/s16101706

**Published:** 2016-10-15

**Authors:** Hao Chen, Xiaoyun Xie, Wanneng Shu, Naixue Xiong

**Affiliations:** 1 College of Computer Science and Electronic Engineering, Hunan University, Changsha 410082, China; samxcode@hotmail.com; 2 College of Computer Science, South-Central University for Nationalities, Wuhan 430074, China; shuwanneng@whu.edu.cn; 3 Department of Computer Science, Georgia State University, Atlanta, GA 30302, USA; dnxiong@ieee.org

**Keywords:** enhanced living environments, big data, recommendation filter model, smart home, Internet-of-Things

## Abstract

With the rapid growth of wireless sensor applications, the user interfaces and configurations of smart homes have become so complicated and inflexible that users usually have to spend a great amount of time studying them and adapting to their expected operation. In order to improve user experience, a weighted hybrid recommender system based on a Kalman Filter model is proposed to predict what users might want to do next, especially when users are located in a smart home with an enhanced living environment. Specifically, a weight hybridization method was introduced, which combines contextual collaborative filter and the contextual content-based recommendations. This method inherits the advantages of the optimum regression and the stability features of the proposed adaptive Kalman Filter model, and it can predict and revise the weight of each system component dynamically. Experimental results show that the hybrid recommender system can optimize the distribution of weights of each component, and achieve more reasonable recall and precision rates.

## 1. Introduction

**Figure 1:**
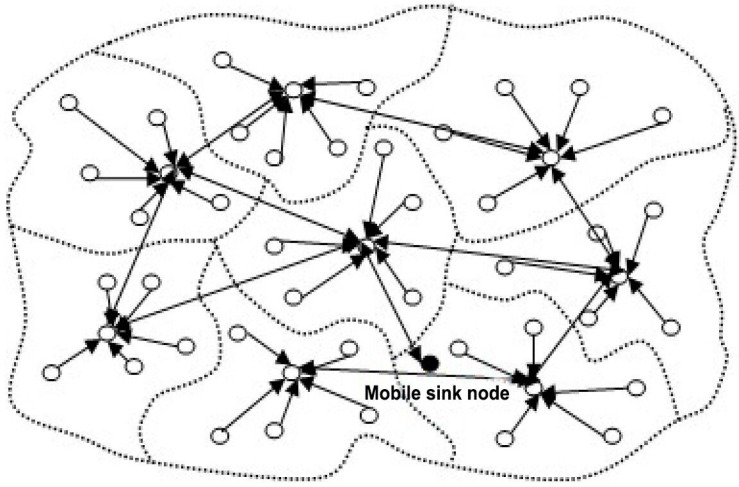
Data forwarding in an ELE.

Recently, multisensory big data analytics and smart homes are playing a crucial role for developing Enhanced Living Environments (ELEs). By being enriched with several sensing capabilities and communication interfaces, a smart environment can be built to improve the quality of life effectively for the elderly or people with special requirements in ELEs. However, there is a major challenge for capturing or recording, storage, searching, correlating, transferring, sharing, and analyzing the huge amounts of data in ELEs owing to the characteristics of multisensory data, for instance, uncertainty, unpredictability or massiveness. Recent advancements in the fields of sensing computing, wireless communication, wearable computing and ubiquitous network environments have paved the way for the emergence of the Internet-of-Things (IoT) paradigm, which aims to connect and network trillions of smart devices, capable of sensing and interacting with the physical world [1]. Figure 1 shows the data forwarding in an ELE and it indicates the transformation between the dormant and active states of sensor nodes. As a result, the energy consumption of nodes can be optimized so as to significantly prolong the network lifetime.

In addition, nowadays smart homes have attracted more and more attention with the rapid development of technologies and applications of the IoT. A smart home (i.e., home automation) consists of a suite of hardware devices which may associate wireless sensor networks with the home environment. By being operated remotely, automatically or scheduled, it meets the requirements of user convenience, safety and efficiency [2]. Provisioning autonomous and intelligent interactions with the environment requires empowering conventional sensor networks with other emerging technologies, such as mobile hybrid recommender systems, wearable devices and mobile nodes, which are collectively referred to as emerging sensor networks [3,4]. 

**Figure 2:**
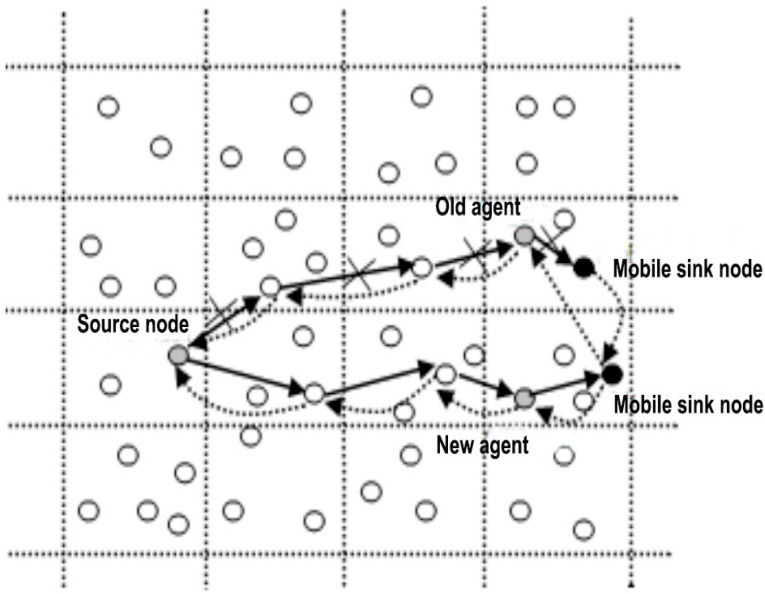
Mobile sink node in the IoT.

Due to the progress in user mobility applications and the rapid popularization of wearable devices, how to provide effective wearable computing and personal recommendations in complicated conditions has become a challenging issue, such as when users are situated in different locations, with various user behaviors and ubiquitous network environments. The increasing popularity of wearable computing sensing boosting devices, such as Google Glass, watches, helmets, shoes, etc., results in potential demands and opportunities for ubiquitous computing via wireless and mobile devices [5,6]. Meanwhile, mobile recommendation technology helps users to extract information which meets their requirements automatically and effectively from the vast ocean of available sensor information. In emerging sensor networks, the prime objective of the topology control techniques is to sustain coverage while ensuring network connectivity and energy conservation. Along with the verging evolution of conventional sensor networks to the IoT, some novel approaches and algorithms are needed to support the above requirements [7,8,9]. Figure 2 shows a mobile sink node in the IoT.

In this paper, a novel hybrid model is presented based on the optimal regressive features of the Kalman Filter. By learning from the training data, the system parameters can be obtained for the Kalman Filter mathematically. Next, the weight of the different component is set through analysis and comparison of the predictions and tangible results. We perform an experiment in a real practical environment, and our algorithm is compared with the item-based collaborative filtering algorithm and content-based algorithm. The experimental results show that the proposed hybrid recommendation algorithm has a positive significant impact on the quality of recommendations.

The rest of paper is organized as follows: Section 2 presents a brief introduction about wireless sensor networks, contextual item-based collaborative filtering recommender systems, contextual content-based recommender systems, hybrid recommender systems and the Kalman Filter model. In Section 3, we review the weaknesses and shortcomings of the traditional hybrid recommendation algorithms and propose a weighted hybrid model based on the Kalman Filter with the optimal estimation feature. In Section 4, the weighted hybrid recommendation algorithm is proposed. The results of the experiments and the analysis of results will be given in Section 5. In Section 6, we draw our conclusions and outline potential future work.

## 2. Related Works

Emerging sensor networks combined with sensor computing, wearable computing, mobile computing, wireless communications and other technologies, have been widely used in the fields of smart cities, smart homes and so on. Although smart homes can provide people great convenience, they are mostly equipped with very complicated closed configurations and user interfaces [10,11]. The inhabitants living in such a smart home have to waste a great amount of time to set and operate these complicated devices and software suites [12,13]. To simplify the user interface and operation, some researchers and engineers have taken advantage of recommender systems to solve this problem.

Rasch [14] proposed an unsupervised hybrid recommender system, which learns the habits of inhabitants automatically and builds much simpler user interfaces. Besides, it can predict what the inhabitant might want to do currently and highlights the most interesting choices available. Vavilov et al. [15] offered a “light” recommendation algorithm for healthcare applications, which is effective, cheap and flexible enough to recommend activities for users and easily adapted to the healthcare goals.

**Figure 3:**
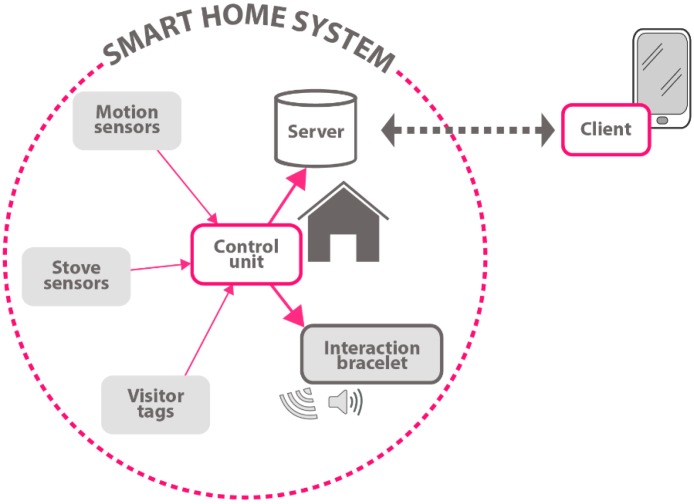
The view of a smart home.

The boom of emerging sensor networks, especially wireless sensor networks, has provided a strong opportunity for smart homes in recent years. Besides, when people are located in the wireless sensor network, abundant contextual information will be obtained for the current environment from various kinds of sensors, such as time, location, temperature, device status, pressure, humidity, and so on [16,17]. Figure 3 shows the view of smart home system. This contextual information is very useful in that it can help us to better analyze the mobility and activity of the inhabitants and even predict what people want to do in the next time period.

The user interfaces and configurations of smart homes are very complicated and inflexible, which results in too much time-consumption for determining and completing the expected actions of users. Furthermore, the problem of information overload is already inevitable in a ubiquitous network scenario. Many researchers have devoted themselves to finding better ways to help users to filter useless information and retrieve valuable contents. Individualization recommendation technology is regarded as an effective and practical approach to solve the information overload problem, especially after the collaborative filtering recommendation algorithm being put forward by Goldberg and others [18]. Although recommendation systems provide users with suggestions for a variety of items from mass data sources, the technique has many disadvantages (e.g., cold-start problems, data sparsity problems).

Due to the complexity of wireless sensor networks, it is hard for a single context-aware recommender system to produce precise recommendations. Many researchers have proposed hybrid recommendation systems which combine two or more techniques to achieve some synergy between several recommendation methods and customize the responses to every user [19]. There are already a lot of applications and experiments that make us understand that hybrid recommender systems can profitably improve the effectiveness of the recommendation process, and most correlation researches are about some hybrid of content-based and collaborative filtering recommendations [20]. 

Mark C. et al. [21] proposed a hybrid approach that combines user profiles which were extracted from the history with the collaborative filter technique, which enhances the effectiveness of prediction results by using a weighted average. However, the accuracy of the predictions is affected mostly by the collaborative filtering prediction, and occasionally the weight value is not precise. Jonathan G. et al. [22] come up with a linear-weighted hybrid framework for making recommendations in social annotation systems, and experiments were conducted using six real-world datasets to prove that the recommender system was more effective and flexible. Gong et al. [23] extracted information from social networks related to users, which can be integrated into a collaborative filter to improve the performance of the system. Fatemeh et al. [24] applied the weighted hybrid recommendation for heterogeneous networks which controls the meta-path to determine which components to include in a hybrid. Domingus et al. [25] proposed a hybrid recommendation model that mixes a blacklisting mechanism with the limitation that the idea is established on the long tail features of music. Lai et al. [26] combined content filtering, neighborhood-based collaborative filter and latent factor model algorithms together using a linear model to achieve high accuracy, but the complexity of the model is too high. Many researchers have improved the performance of weighted hybrid recommendations based on collaborative filters and content-based algorithms. In this task how to optimize the weight is one key for the weighted hybridization, and that is the topic to which we pay more attention in this paper.

**Figure 4:**
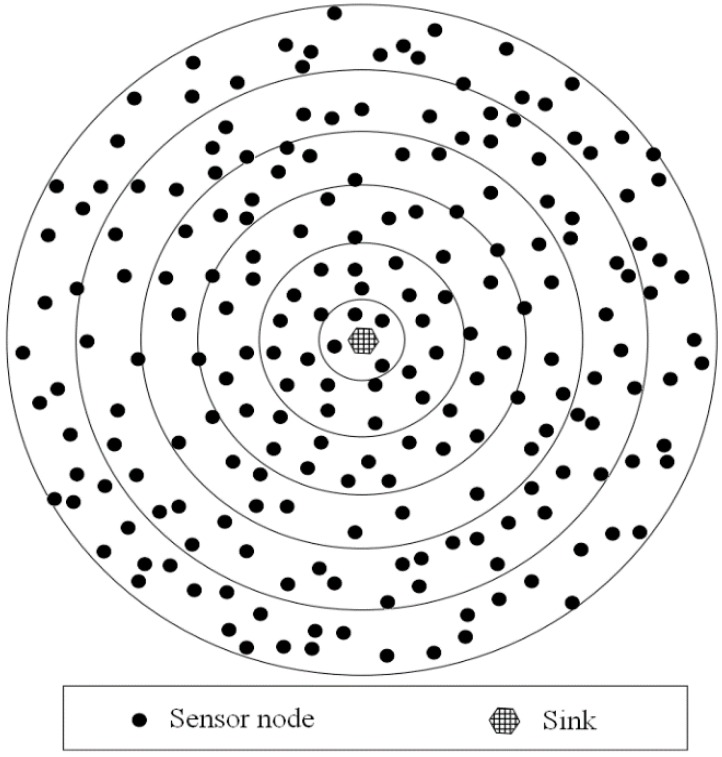
The deployment of sensors in emerging sensor networks.

Wireless sensor networks (in particular wireless sensor and actuator networks) consist of plenty of specialized sensor nodes, which are spatially dispersed and have a dedicated communication infrastructure. By monitoring the physical or environmental conditions at diverse locations, the sensors measure, collect and process information about the target area, such as light, sound, location, pressure, temperature, humidity, and so on. With the progress of technology, sensors have already become small, cheap, lightweight, and portable. Despite limited processing and computing ability, each tiny sensor is equipped with transducer, microcomputer, and transceiver. After sensing and collecting information from the target area of interest, the sensor node should transmit the collected data to users or other devices using certain wireless communication techniques [27]. The deployment of sensors in emerging sensor networks is shown in Figure 4.

Some wireless sensor network communication protocols have been widely applied, such as WiFi, Bluetooth, ZigBee, and Wireless HART. Due to the limited capacity of batteries and unrechargable environment, an effective power management scheme must be considered. In wireless sensor networks, a large number of tiny nodes are deployed randomly and capable of assembling and configuring by themselves to withstand harsh environmental conditions and ease of use. In the early design stages, wireless sensor networks were mainly focused on specifically promoting the effectiveness of military action. But nowadays they have been applied in many fields, such as target tracking and identification, biological health monitoring, air pollution monitoring, smart homes, medical applications, and industrial automation [28], etc. In the smart home based on wireless sensor networks, a wide range of automation devices like entrance management components are provided, and inhabitants who lived in the smart homes can manage and control the systems locally or remotely.

**Figure 5:**
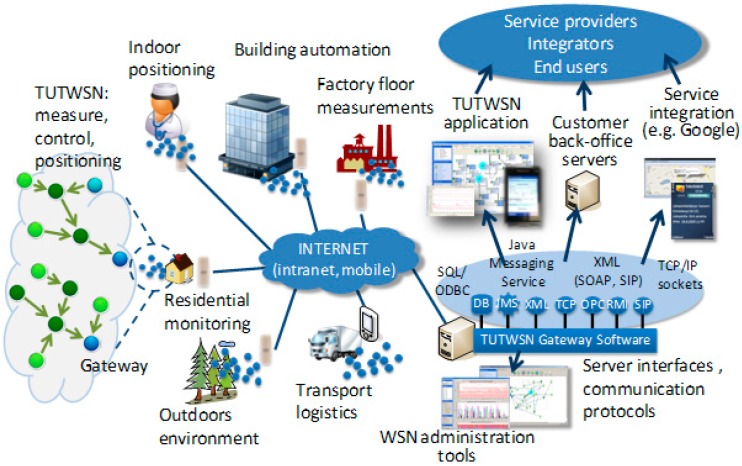
The architecture of a typical wireless sensor network.

A wireless sensor network consists of small, low-cost, and low-energy sensor nodes that cooperatively monitor physical quantities and control actuators, and thousands of randomly deployed nodes can operate autonomously to form a multi-hop topology. In this point, the numerous self-configurable sensor nodes should run in an adaptive manner with their environment and execute sensing, computing, actuating, and communication tasks. The architecture of a typical wireless sensor network is illustrated in Figure 5.

Context describes the information which characterizes the situation of the entity in the ubiquitous computing. Emerging sensor networks can provide us much abundant context information. The widely used context usually reflects the physical environment (e.g., location, time). However, some contexts can include other types of data, for instance, information about the user (the user’s habits, bio-physiological conditions, etc.), physical conditions (noise, light, temperature, etc.), and social environment (social interaction, co-location with other users, etc.) [29]. In this system, we observe the context of information about the user and physical conditions, and the context can be given as a vector of different context types [30]:

(1) $$C=(C_{1},C_{2},\cdots C_{z})$$

where *C_t_* (*t* ∈ 1,2,…,*z*) is a context type, such as location, time, temperature, and so on.

The Pearson Correlation Coefficient method is adopted to measure the simility between two different contexts *Sim*(*x,y*). Then, we have:

(2) $$Sim(x,y)=\frac{n\sum xy-\sum x\sum y}{\sqrt{n\sum x^{2}-{\left(\sum x\right)}^{2}}\times \sqrt{n\sum y^{2}-{\left(\sum y\right)}^{2}}}$$

where *x* and *y* are two different contexts.

Based on Equation (2), we define the number of operations or activities done by the user *u* on the device *i* in the context *x* as *r_u,x,i_*:

(3) $$rel(x,y,i)=k\frac{\sum_{u\in U}\left(r_{u,i,x_{t}}-{\bar{r}}_{i})\times (r_{u,i,y_{b}}-{\bar{r}}_{i}\right)}{\sigma_{x}\times \sigma_{y}}$$

where *k* is a coefficient that is used to adjust the sensity of the relavance, and ${\bar{r}}_{i}$ is the average number of operations. *U* represents the set of all users in the system. *σ_x_*, *σ_y_* are the standard deviations for the two contexts. *rel*(*x,y,i*) returns the relevance of the two context values in *C* over all the number of operations done by users. To get a better and clearer result, we can incorporate this relevance feature into the similarity calculations. Besides, context information usually involves in privacy problems. For this reason, in our recommender system, the information data is encrypted.

For most item-based collaborative filtering algorithms the similarity (conditional probability-base similarity, cosine-based similarity, etc.) between different items can be calculated by analyzing historical use or purchasing data, which is usually presented as a user-item matrix. Then, a recommendation list which is sorted by using some interest measure for items will be derived in a Contextual Item-based Collaborative Filtering (CICF) recommender system [31]. The main idea of collaborative filtering is to predict the items that people will buy or prefer according to what they liked or bought in the past [32,33]. The process of the contextual item-based collaborative filter algorithm has three steps:

1. Disposal of the historical data about the users and items and building of the user-item matrix.
2. Calculation of the similarity between each pairs of items, and building of an item-item similarity matrix.
3. Calculation of the current user’s location-aware taste for each item by the item-item similarity matrix and the user’s historical record, and choosing the most interesting items to generate the recommendations.

Different from the case of desktop calculation, there is more abundant context information in the wireless sensor environment, and the recommendation systems that can achieve better performance benefits from the context, for example, location or time [34]. CICF is also based on the idea of collective intelligence, and brings contextual information into the similarity of items and collaborative filtering model. The key of CICF is that calculates preference similarities of items in the context condition. 

Chen proposed a context-aware collaborative filtering recommender system that integrates contextual information of items and user-context information into the collaborative filtering, and represents the similarity calculation method based on the item-context correlation coefficient [35]. Based on their work, we also consider the distance between the active use and items using a similar method.

The probability of the operation *i* that the user *u* does in the context *c* can be defined as *P*(*u*,*i*,*c*), and it can be formulated as:

(4) $$P(u,i,c)=\frac{n_{i,c}}{\log(1+\sum_{C}n_{i}^{\left(u\right)})}\sum_{x\in C}\sum_{j\in N(U)}S\text{im}(x,c,j)r_{u,j,c}$$

where *N*(*U*) represents the set including all the operations that the user *u* ever did, *n_i_*_,*c*_ is the number of operations *i* done by current user in the context *c*, $n_{i}^{\left(u\right)}$ is the total number of operations *i* done by all users, *r_u_*_,*i*,*c*_ is the probability of user doing the operation *i* in the context *c*.

A Contextual Content-Based (CCB) recommender system in one which compares the contents of items and the profiles of users. Its main idea is to recommend items whose content attributes are similar to those of the items that users ever liked or bought before. There are many ways to present the items. In this paper, a TF-IDF model is applied to assign different weight to the item attributes, which is regarded as a vector space with a given weight. The TF-IDF model is the product of two statistics: term frequency and inverse document frequency. Then it can be calculated using the following equation:

(5) $$d_{i}=\left\{\left(e_{1},w_{1}\right),\left(e_{2},w_{2}\right),\left(e_{3},w_{3}\right)\cdots \right\}$$

In which, *e_i_* is the certain keyword in the attributes of the item *i*, and *w_i_* is the weight of corresponding keyword. The process of the contextual content-based recommendation method also includes four steps:

1. Extract the item character to build the model for presenting the item.
2. Use the features of items which the user ever liked or bought to create the user preference.
3. Generate a list of recommended items for the user by comparing the relevancy of the user preference and items.

A contextual content-based recommender system brings contextual information into the content-based recommendation algorithm, emphasizing the matching rate of user preferences, context and item properties. Through mining user preferences for different item properties in different contexts, the algorithm combines with the property descriptions of each specific item to predict potential user preferences and generate recommendations. 

Chihiro et al. constructed a Bayesian network model to acquire user preferences for an item set with respect to different contexts (prior probability). Next, by calculating the post-probability of user potential preference to unseen or never-bought items with specific property vectors in a specific context environment, *N* items of higher preference to users can be extracted and recommended [36]. In our system, we also integrate the Bayesian network model into the content-based recommendation algorithm, similar to Chen’s algorithm.

## 3. Discrete Kalman Filter Model

### 3.1. Hybrid Recommender System

Either item-based collaborative filtering or different content-based methods have their own weakness and strengths. Hybrid recommender systems produce their output by combining various methods organically [37]. They can choose different strategies according to the particular case, and avoid the defects of the recommendation methods to improve the recommendation performance and provide more valuable recommendations.

In general, there are several hybrid ways: weighted, switching, mixed, feature combination, feature augmentation, cascade and meta-level [38]. Weighted is about the output of the different recommender system components combined using a linear weight scheme. 

By using a linear weight formula, the weighted hybridization produces the results by combining the output of the two or more components, and the simplest weighted hybridization can be represented by the following equation:

(6) $$P=\sum_{i=1}^{n}c_{i}P_{i}$$

where *P_i_* is the result generated by the recommendation component *i*, and *c_i_* is the weight of the component *i*.

However, the above model does not consider takes into account the individual characteristics rather than the holistic character of weights. To avoid these defects, we use the following model to calculate and assign weights:

(7) $$P(u,i)=\sum_{m\in M}c(m,u,i)\times P(m,u,i)$$

where *P*(*u,i*) is the degree of interest of user *u* in item *i*. *P*(*m*,*u,i*) is the interest degree of the user *u* to the item *i* produced by the recommender component m. *c*(*m*,*u,i*) is the weight of user *u* to the item *i* produced by the recommender component *m*. *M* represents all of recommendation components in the recommender system. 

### 3.2. Discrete Kalman Filter Model

Kalman filtering, usually referred to the discrete Kalman Filter, is a linear quadratic estimation algorithm that tries to produce an unbiased estimate of the state of a dynamic uncertain system [39]. If all samples of the system noise demonstrate a Gaussian distribution, a Kalman Filter can minimize the mean square error of the estimated parameters, which can be proven as an optimal estimator. 

The prediction and revision process of a Kalman Filter is relatively fast because of not keeping any history other than the previous estimate state. As a result, it can run in real time and doesn’t need much memory space. It is also easy to implement in practical applications. The Kalman Filter has numerous applications in technology and is widely employed in the fields of the communication, GPS navigation, robot vision and image painting [40,41,42]. 

The discrete Kalman Filter is mainly applied to estimate the state of a discrete-time controlled system [43,44]. The controlled process of a discrete Kalman Filter can be represented as two linear stochastic difference equations [45,46,47]. *x_k_* is the system state at the time *k*, which can be estimated by the following Equation (8):

(8) $$x_{k}=A_{k}x_{k-1}+Bu_{k}+w_{k-1}$$

where *u_k_* is the system control variable, the variables *A_k_* and *B_k_* represent the parameters of the system state model, *w_k_* is the process noise.

With an observed equation:

(9) $$z_{k}=H_{k}x_{k}+v_{k}$$

In which, *z_k_* is the system measurement, *H_k_* is the model parameter of the observed system, *v_k_* is the measurement noise.

In the above Equations, both the system process noise and the measurement noise are assumed as Gaussian white noises. *w_k_* is a Gaussian distribution with mean 0 and standard deviation *Q_k_*. Here, *Q_k_* represents the process noise covariance, which is a non-negative definite matrix. *w_k_* is a Guassian distribution with mean 0 and standard deviation *R_k_*, *R_k_* represents the measurement noise covariance, which is a positive definite matrix:

(10) $$\left\{ \begin{array}{l} p(v)\sim N(0,R_{k})\\ p(w)\sim N(0,Q_{k}) \end{array}\right.$$

**Lemma** **1.** *assuming that P_s_ is a constant, it will have optimal solution* $P_{r}^{*}=\{P_{r_{1}}^{*},P_{r_{2}}^{*},...,P_{r_{M}}^{*}\}$ *for Equation* *(10). It satisfies*:

(11) $$\sum_{m=1}^{M}P_{r_{m}}^{*}\leq \sum_{m=1}^{M}P_{r_{m}}$$

**Proof.**  For *λ*(*P*), if we can prove it is a concave function, the global optimal point is the only optimal point. By taking the derivative of *λ*(*P*) to $P_{r_{m}}$, we have:

(12) $$\frac{\partial \lambda }{\partial P_{r_{m}}}=\frac{G_{r_{m}d}G_{sr_{m}}P_{s}\left(G_{sr_{m}}P_{s}+\sigma^{2}\right)}{{\left(P_{r_{m}}G_{r_{m}d}+P_{s}G_{sr_{m}}+\sigma^{2}\right)}^{2}\sigma^{2}}>0$$

where $G_{r_{m}d}={\left|h_{i1mk}\right|}^{2}$ and $G_{sr_{m}}={\left|h_{i3mk}\right|}^{2}$.

(13) $$\frac{\partial^{2}\lambda }{\partial^{2}P_{r_{m}}}=-G_{r_{m}d}G_{sr_{m}}\frac{2G_{r_{m}d}G_{sr_{m}}P_{s}^{2}+2\sigma^{2}G_{r_{m}d}P_{s}}{{\left(P_{r_{m}}G_{r_{m}d}+P_{s}G_{sr_{m}}+\sigma^{2}\right)}^{3}\sigma^{2}}<0$$

(14) $$\frac{\partial^{2}\lambda }{\partial P_{r_{m}}P_{r_{n}}}=0,(m\neq n)$$

□

Since *P_s_* is a constant, *λ*(*P*)’s Hessian matrix must be a negative definite matrix. Therefore, *λ*(*P*) is a concave function. 

Next, the optimal solution can be obtained by Lagrangian methods, and the Lagrange function is defined as follows:

(15) $$L(P,\gamma )=N\sum_{m=1}^{M}P_{r_{mk}}-\gamma \left(1+\lambda_{ik}^{D}+\sum_{r\in \{r_{1},r_{2},...,r_{M}\}}\lambda_{ik}^{r}-\lambda_{0}\right)$$

where *γ* is the Lagrange multiplier and $P=\left\{P_{rm1},P_{rm2},\ldots ,P_{rmN}\right\}.$ Then, by taking the derivative of *L*(**P**,*γ*) to $P_{r_{mk}}$ and equating it to zero, we have:

(16) $$\frac{\partial L(P,\gamma )}{\partial P_{r_{mk}}}=N-\gamma \frac{|h_{i2mk}{|}^{2}|h_{i3mk}{|}^{2}P_{sk}\left(|h_{i3mk}{|}^{2}P_{sk}+\sigma^{2}\right)}{{\left(P_{r_{mk}}|h_{i2mk}{|}^{2}+P_{sk}|h_{i3mk}{|}^{2}+\sigma^{2}\right)}^{2}\sigma^{2}}=0$$

By simplifying and some manipulations to Equation (16), we estimate $P_{r_{mk}}$ as:

(17) $$P_{r_{mk}}=\sqrt{\frac{\gamma |h_{i3mk}{|}^{2}P_{sk}\left(|h_{i3mk}{|}^{2}P_{sk}+\sigma^{2}\right)}{N|h_{i2mk}{|}^{2}\sigma^{2}}}-\frac{P_{sk}|h_{i3mk}{|}^{2}+\sigma^{2}}{|h_{i2mk}{|}^{2}}$$

According to Equation (17), *λ*(**P**) can be written as:

(18) $$\lambda (P)=1+\frac{P_{sk}|h_{i1mk}{|}^{2}}{\sigma^{2}}+\sum_{m=1}^{M}\frac{|h_{i2mk}{|}^{2}|h_{i3mk}{|}^{2}P_{sk}P_{r_{mk}}}{\left(P_{r_{mk}}|h_{i2mk}{|}^{2}+P_{sk}|h_{i3mk}{|}^{2}+\sigma^{2}\right)\sigma^{2}}=\lambda_{0}$$

In practical application, it is reasonable to assume that $P_{sk}{\left|h_{i3mk}\right|}^{2}\sigma^{2}$. Otherwise, the relay node will be excluded by the source node at the beginning. Hence, we can assume that $P_{sk}{\left|h_{i3mk}\right|}^{2}+\sigma^{2}\approx P_{sk}{\left|h_{i3mk}\right|}^{2}$ and substitute it and Equations (17) and (18), giving:

(19) $$\sqrt{\gamma }=\frac{\sigma P_{sk}\sum_{m=1}^{M}\frac{|h_{i3mk}{|}^{2}\sqrt{N}}{\left|h_{i2mk}\right|}}{P_{sk}|h_{i1mk}{|}^{2}-\sigma^{2}\left(\lambda_{0}-1\right)+P_{sk}\sum_{m=1}^{M}|h_{i3mk}{|}^{2}}$$

For the sake of notation simplicity, we define D=|hi1mk|2+∑n=1M|hi3mk|2, Am=|hi3mk|2|h12mk|2 and Bm=|hi3k|2|h12k|2. By substituting them into Equation (19), the optimal power consumption of relay node *r_m_* is given by:

(20) $$P_{r_{mk}}^{*}=\frac{A_{m}P_{sk}^{2}}{\left(DP_{sk}-\sigma^{2}\left(\lambda_{0}-1\right)\right)}\sum_{n=1}^{M}A_{n}-B_{m}P_{sk}$$

In this section, we have briefly introduced the hybrid recommender system and the discrete Kalman Filter model. Because of the complexity of current hybrid methods, the hybrid recommendation is hard to apply to real environments, and the Kalman Fitler can help us reduce the complexity.

## 4. The Weighted Hybrid Recommendation Algorithm

Our hybridization approach is based on combining collaboration filtering component prediction with content-based component prediction. The key of weighted hybridization is that the weight of each component can be precisely assigned and predicted using a Kalman Filter, which can optimally estimate the system state with the measurements. 

Focusing on the hybrid context-aware recommender system for predicting users’ preference in the contextual environment, a Weighted Hybrid Recommendation Algorithm based on Kalman filter (WHRA-KF) is introduced. This strategy aims to design a novel weighted hybrid recommender system, which can understand the activities of users more clearly and unambiguously derived from much abundant contextual information in the wireless sensor network environment. In addition, the algorithm can predict and revise the weight of each system component dynamically by taking advantages of the optimum regression feature of the Kalman Filter model under a variety of different requirements. The proposed algorithm applies the Kalman Filter model to predict and revise the weight in emerging sensor networks. As the collaboration filter and content-based recommendation components, we will use the two common algorithms based on the work of other researchers, so we can pay our attention on the way of the weighted hybrid recommendation based on Kalman Filter model.

### 4.1. The Hybrid Recommendation Model

We will model the process for predicting the weight in accordance with the framework of the Kalman Filter. For the user *u* in the recommended time *i*, we assume that *x*(*i*,*u*) is the ratio of the content-based component weight *w_CCB_*(*i*,*u*) to the item-based collaborative filtering component weight *w_CICF_*(*i*,*u*):

(21) $$x(i,u)=\frac{w_{CCB}(i,u)}{w_{CICF}(i,u)}$$

If we want to make use of the optimal estimation of the Kalman Filter reasonably, the first step is to build a reasonable and precise state transition model. There is an assumption that the ratio *x*(*i*,*u*) is stochastic and linear in any given time for the user *u* according to the notion that the user’s habit changes display some regularity.

For convenience, we will omit the index *u* in the following section. There is a linear relation *f* between *x*(*i*) and *x*(*i* *−* 1):

(22) x(i)=x(i−1)+αv(i−1)

(23) v(i)=x(i−1)−x(i−2)

where *α* is the system parameter used to enhance or slow the speed of change of the ratio and its value can be obtained by multiple tests. Therefore, the system state of the Kalman Filter can be represented as:

(24) $$x_{k}={\left[x(k),v(k)\right]}^{T}$$

We collect the counts of last recommendations of both components the user really liked or bought, then we calculate the ratio of the numbers of two components successfully recommended. The a posteriori estimation is also being represented in the observation *z_k_*:

(25) $$x_{k}=x_{k-1}^{-}$$

The model parameters *A_k_*, *H_k_* can be deduced by the linear relation of Equations (12) and (13):

(26) $$A_{k}=\left[\begin{array}{cc} 1 & \alpha \\ 0 & 1 \end{array}\right],H_{k}=\left[\begin{array}{cc} 1 & 0 \end{array}\right]$$

In addition, we assume that the first matrix of the predictive error covariance is:

(27) $$P_{0}=\left[\begin{array}{cc} 1 & 0\\ 0 & 1 \end{array}\right]$$

The Kalman Filter process includes two steps: prediction and correction. The prediction process includes the prediction for the a priori state estimate and estimate covariance. The correction process includes innovation or measurement residual, residual covariance, optimal Kalman Gain, and updating the state estimate and estimate covariance.

The prediction for the preliminary weight is given as

(28) $${\hat{x}}_{k|k-1}=A{\hat{x}}_{k-1}+Bu_{k}$$

The prediction for the a priori error covariance is given as:

(29) $$P_{k|k-1}=AP_{k-1}A^{T}+Q_{k}$$

The process of correction is to revise “Kalman Gain”, we have:

(30) $$\frac{\partial tr(P_{k})}{\partial K_{k}}=-2(H_{k}P_{k}^{k-1})+2K_{k}S_{k}=0$$

(31) $$K_{k}S_{k}={\left(H_{k}P_{k|k-1}\right)}^{T}=P_{k|k-1}H_{k}^{T}$$

(32) $$S_{k}=\mathrm{cov}(z_{k}-H_{k}{\hat{x}}_{k|k-1})$$

where *S_k_* is the innovation covariance. From the above Equations (19)–(21), *K_k_* can be calculated as:

(33) $$K_{k}=P_{k|k-1}H^{T}{\left(HP_{k|k-1}H^{T}+R\right)}^{-1}$$

Then, the a posteriori state estimate for the weight can be replaced as:

(34) $${\hat{x}}_{k}={\hat{x}}_{k|k-1}+K_{k}(z_{k}-H{\hat{x}}_{k|k-1})$$

Consequently, the a posteriori error covariance matrix can be updated, and we have:

(35) $$P_{k|k}=(I-K_{k}H)\mathrm{cov}(x_{k}-{\hat{x}}_{k|k-1})(I-K_{k}H_{k})+K_{k}\mathrm{cov}(v_{k})K_{k}^{T}$$

Equation (25) is better known as the “Joseph form” of the covariance update Formula. It turns out that if *K_k_* is the optimal Kalman Gain, this can be simplified as:

(36) $$P_{k}=(I-K_{k}H)P_{k}^{-}$$

The Kalman Filter is a recursive estimator in that it actually makes an iterative calculation for the state ${\hat{x}}_{k}$ and its error covariance *P_k_*.

### 4.2. Optimal Adaptive Factor

Under normal circumstances, we usually assume that the process noise *w_k_* and the measurement noise *v_k_* are Gaussian white noises with mean of 0 and known covariance *Q_k_*, *R_k_*. Through trial and error, the statistical properties of *Q_k_* can be obtained, but the statistical properties of *R_k_* are unknown, especially in a complicated environment. Furthermore, both of them are not invariable and change in accordance with the conditions of the application environment. Thus, we can make use of the measurement information to adjust the noise excitation of the Kalman Filter, and the system model should be continuously refined if we do this.

To enhance the adaptability, a maximum posterior estimator is developed to estimate the statistical properties of the process noise and the measurement noises dynamically, which is one of the evolutionary methods based on the Sage adaptive filtering algorithm [48]. With the help of Sage adaptive filtering, we can work out:

(37) $${\hat{q}}_{k}=(1-\frac{1}{k}){\hat{q}}_{k-1}+\frac{1}{k}({\hat{x}}_{k}-A_{k}{\hat{x}}_{k-1})$$

(38) $${\hat{r}}_{k}=(1-\frac{1}{k}){\hat{r}}_{k-1}+\frac{1}{k}(x_{k}-H_{k}{\hat{y}}_{k-1})$$

(39) $${\hat{Q}}_{k}=(1-\frac{1}{k}){\hat{Q}}_{k-1}+\frac{1}{k}(P_{k}-A_{k}P_{k}A_{k}^{T})$$

(40) $${\hat{R}}_{k}=(1-\frac{1}{k}){\hat{R}}_{k-1}+\frac{1}{k}(x_{k}x_{k}^{T}-H_{k}P_{k}H_{k}^{T})$$

In which, ${\hat{q}}_{k}$ is the a posteriori estimation of the mean of the process noise, and ${\hat{Q}}_{k}$ is the a posteriori estimation of the process error covariance matrix. It is the same for ${\hat{r}}_{k}$ and ${\hat{R}}_{k}$ as well. The above four formulas help us estimate the statistical properties of the process and measurement noises.

Moreover, the variation of the residual ${\hat{y}}_{k}$ can be measured to determine whether the parameters *Q_k_* and *R_k_* should be modified or not. The residual ${\hat{y}}_{k}$ is the difference between the real measurement value and the estimated measurement value in the Kalman Filter model at the time *k*:

(41) $$y_{k}=z_{k}-H_{k}{\hat{x}}_{k|k-1}$$

The residual *y_k_* reflects the level of dependency of the system model on the measurements. As above, the residual is white noise with mean 0, if the model is accurate enough. If the residual ceases to be white noise with mean 0, problems occur with the filter, and further, the Kalman Gain won’t be optimal. 

We use the mean and estimated covariance of the residual in the Kalman Filter to judge the performance of the filtering. Assuming *n* represents the statistical number over a period of time, the mean of the residual can be calculated by the following equation:

(42) $$\bar{y}=\frac{1}{n}\sum_{j=t-n}^{t}r_{j}$$

The covariance matrix of the residual *P_r_* is related to *Q_k_*, *R_k_* which can be calculated by the following formula:

(43) $$P_{r}=H_{k}(EP_{k-1}E^{T}+Q_{k})H_{k}^{T}+R_{k}$$

Then, the posterior estimated covariance of the residual ${\hat{P}}_{r}$ can be calculated as:

(44) $${\hat{P}}_{r}=\frac{1}{n}\sum_{j=t-n-1}^{t}y_{j}y_{j}^{T}$$

Next, the estimation ${\hat{P}}_{r}$ and *P_r_* should be analyzed. When ${\hat{P}}_{r}$ is more and more greater than *P_r_*, and the mean $\bar{y}$ gradually moves away from 0, the filtering becomes increasing unstable, then we adjust the excitation of noises through Equations (29) and (30). Otherwise, we just keep the way of the calculation for the noise *Q_k_*, *R_k_* unchanged. If *P_r_* is close to 0 and mean approximates to 0, then *Q_k_* and *R_k_* should remain unchanged. Otherwise, while both *P_r_* and the mean are far from 0, then $Q_{k}={\hat{Q}}_{k}$ and ${\hat{R}}_{k}=R_{k}$.

Due to the adaptive feature, the noises of the system and measurement impact the real result calculated by the Kalman Filter slightly [49]. We can observe that the Kalman Filter tries to coverage to correct estimations, even if they are poorly estimated. We have performed some rough tests to estimate the covariance matrix of the system noise and measurement noise:

(45) $$Q_{0}=\left[\begin{array}{cc} \frac{1}{2} & 1\\ 0 & \frac{1}{2} \end{array}\right],R_{0}=\left[\frac{1}{10}\right]$$

### 4.3. The WHRA-KF Process 

Only the estimated state from the previous time step and the current measurement are what the Kalman Filter model needs to compute and predict the estimate for the current state [50]. In the following processes, ${\hat{x}}_{k|k}$ represents the a posteriori state estimate at time *k* given observations up to and including at time *k*. $P_{k|k}$ represents the a posteriori error covariance matrix, namely a measure of the estimated accuracy of the state estimate [43]. The main process of the proposed algorithm is as follows:

1. Disposal and input of the user data and item data.
2. Run the CICF and CCB algorithm to generate the initial recommendations.
3. Start the adaptive Kalman filtering process, and predict the weight ratio.
4. According to the stochastic linear relation *f*, calculate the final weights of two components, and generate the final recommendation list.
5. Verify the recommendations, and add the number of recommended items that users really liked or bought. Then calculate the ratio of the above two numbers, and make the ratio the next measurement variable *z_k_*_+1_.

**Figure 6:**
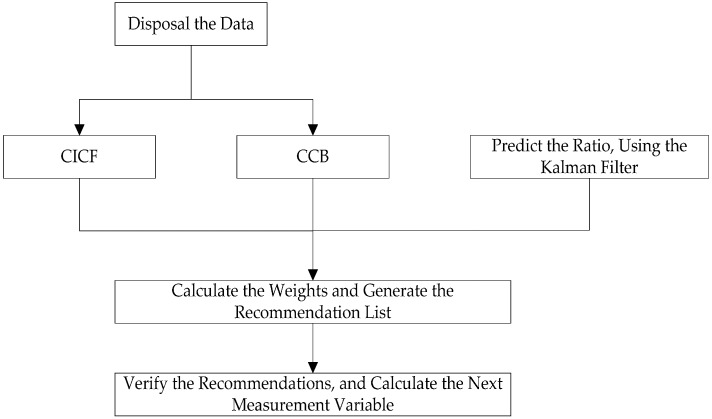
The WHRA-KF process.

This iterative process is continued until the optimal estimation of the system state is obtained at every turn. As time goes on, we can make effective predictions of the weight of the hybrid recommender system and improve the quality of recommendations with the use of the regression optimization features of the Kalman Filter. Figure 6 shows the process of WHRA-KF.

## 5. Experiments and Evaluation

In this section, we explored some simulations to evaluate the effectiveness of our hybrid approach. To test the proposed method empirically, we constructed a smart home at KuTing using emerging sensor networks and performed preliminary experiments with our algorithm using the real dataset from KuTing. Further simulations were also conducted with some traditional recommendation algorithms for the sake of a comparison between their performance and that of the proposed method.

### 5.1. Description of Dataset

**Figure 7:**
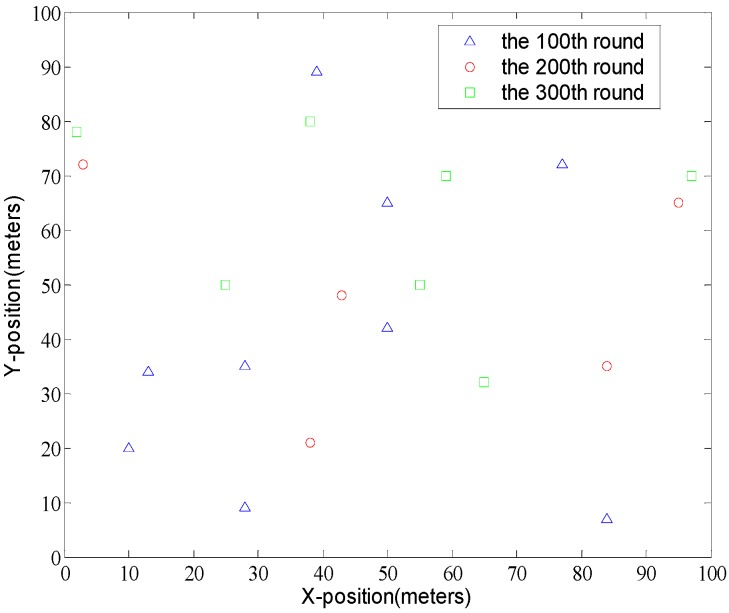
The distribution of cluster heads in WHRA-KF

The experiment dataset is collected from a real practical environment at the KuTing smart home project. It includes the behavioral data of 300 users and the context data from smart homes over a period of 30 days. The smart homes are divided into two different types: small and big. The standard small smart home is equipped with 50 sensors, and the deluxe big smart home is equipped with 100 sensors. Clearly, the more sensors deployed, more context information can be obtained. These smart sensors monitor the status of doors, fridges, TVs, or air conditioners continuously, and plenty of contextual information can be obtained. For example, we collected the data, such as time, temperature, and so on, generated by these sensors every 20 s over a period of 30 days. Human activities are identified as the operation of smart devices, such as opening/closing the front door, cranking up/turning down the air-conditioners and so on. Figure 7 shows the distribution of cluster heads in WHRA-KF.

### 5.2. Evaluation Metrics

To verify the performance of the proposed algorithm, several evaluation indexes (e.g., precision, recall, F-measure and coverage) are considered, which are popular ways of measuring top-*N* recommender systems. For predicting the user’s next activity, it’s necessary for the proposed recommender system to run in real-time and generate the recommendations in time, so we also measure the time cost of the system.

Precision is defined as the proportion of recommendations that are valid recommendations, while Recall is the proportion of valid recommendations that appear in top recommendations [44,45,46]:

(46) $$Precision=\frac{\sum_{u}\left|R(u)\cap T(u)\right|}{\sum_{u}\left|R(u)\right|}$$

(47) $$Recall=\frac{\sum_{u}\left|R(u)\cap T(u)\right|}{\sum_{u}\left|T(u)\right|}$$

Here, the variable *R*(*u*) is the recommendation list for the user *u*, *T*(*u*) represents all items in the test dataset with relation to the user *u*.

The F-measure, namely *F*_1_, is a measure that is the harmonic mean of the precision and recall, which originally was often used in the fields of measuring information retrieval, document classification, and query classification performance, and now is widely used in the recommender system for a summary measure. The term coverage refers to the proportion of items that the recommender system can recommend. The common measure of Coverage is the percentage of all items that are recommended to users during an experiment. The coverage can be represented as:

(48) $$Coverage=\frac{\left|\sum_{u\in U}R(u)\right|}{I}$$

In which, the variable *U* represents all users, and *I* represents all items.

### 5.3. The Analysis of Results

In the experiments, the initial state *x*_0_ just has an impact on the speed of the convergence of the algorithm, so we set the parameter of the state as $x_{0}=\left[\begin{array}{c} 1\\ 0 \end{array}\right]$ and *n* is expressed as the number of sensors in the emerging sensor networks. 

This is due to the fact that in the smart homes the inhabitants usually make use of various software programs embedded in resource constrained mobile phones or wearable devices to control the home devices, and there is not enough space to show more recommendation choices. If the number of choices is more than 10, inhabitants may waste a lot of time to browse all the recommendations, so we have set the range of the recommendation list as 2–10. 

**Figure 8:**
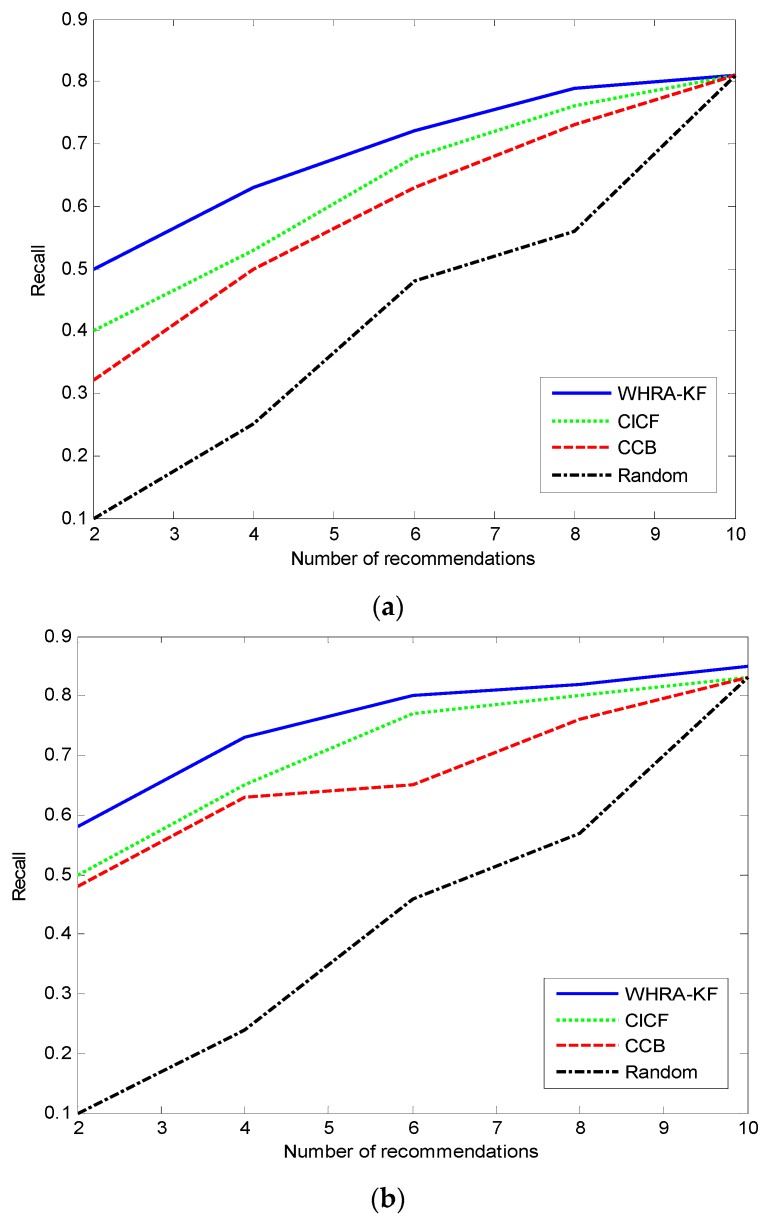
Recall VS number of recommendation for smart home, (**a**) n=50; (**b**) n=100.

Figure 8 shows the Recall values for the different recommendation algorithms. During the experimental phase, the parameters in the standard and deluxe smart homes are set according to the KuTing smart home dataset, respectively. The x-axis denotes the number of recommendations, and the y-axis is the Recall. Figure 8a represents the situation of the standard smart home equipped with 50 sensors, and Figure 8b represents the situation of the deluxe smart home equipped with 100 sensors.

It’s obviously that the value of Recall increases with the number of recommendations for all recommendation algorithms, but the extent of the increase reduces as the number of recommendations increases too. For n=100, when the number of recommendations is 10, the recall achieves the best value of 0.82, that’s to say that 82% of all valid recommendations appear in the top recommendations. On the contrary, the proposed recommender system gives the worst Recall value of 0.57 when the number of recommendations is 2. The phenomenon in the standard smart home is similar.

**Figure 9:**
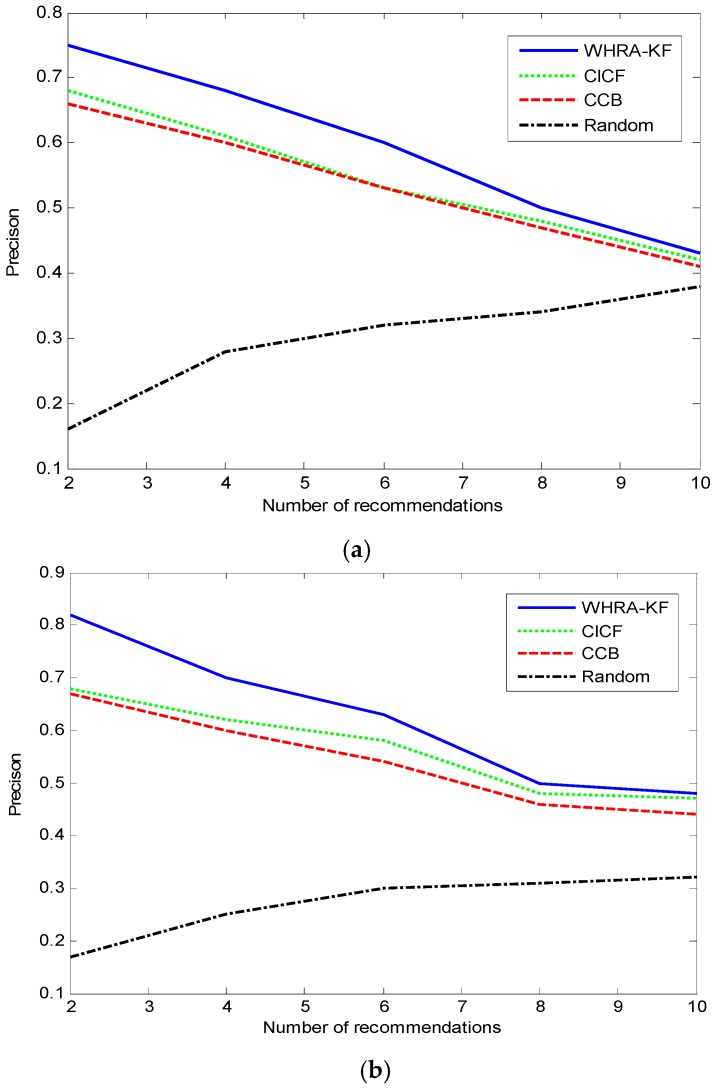
Precision VS number of recommendation for smart home, (**a**) n=50; (**b**) n=100.

Figure 9 shows the comparison of the value of Precision under the same conditions. Unlike the Recall trend, we can see that the value of Precision decreases with the number of recommendations in both smart homes except for the Random recommendation algorithm. The precision value of the Random algorithm increases along with the number of recommendations just the other way. For n=100, when the number of recommendations is 2, the proposed system shows its best performance, then the recall is 0.83 and far surpasses the other algorithms. On the contrary, when the number of recommendations is 10, the Recall value is only 0.48 which is close to the Precision of CICF and CCB. Figure 9 shows that the value of recall decreases along with the number of recommendations for the WHRA-KF, CICF and CCB.

**Figure 10:**
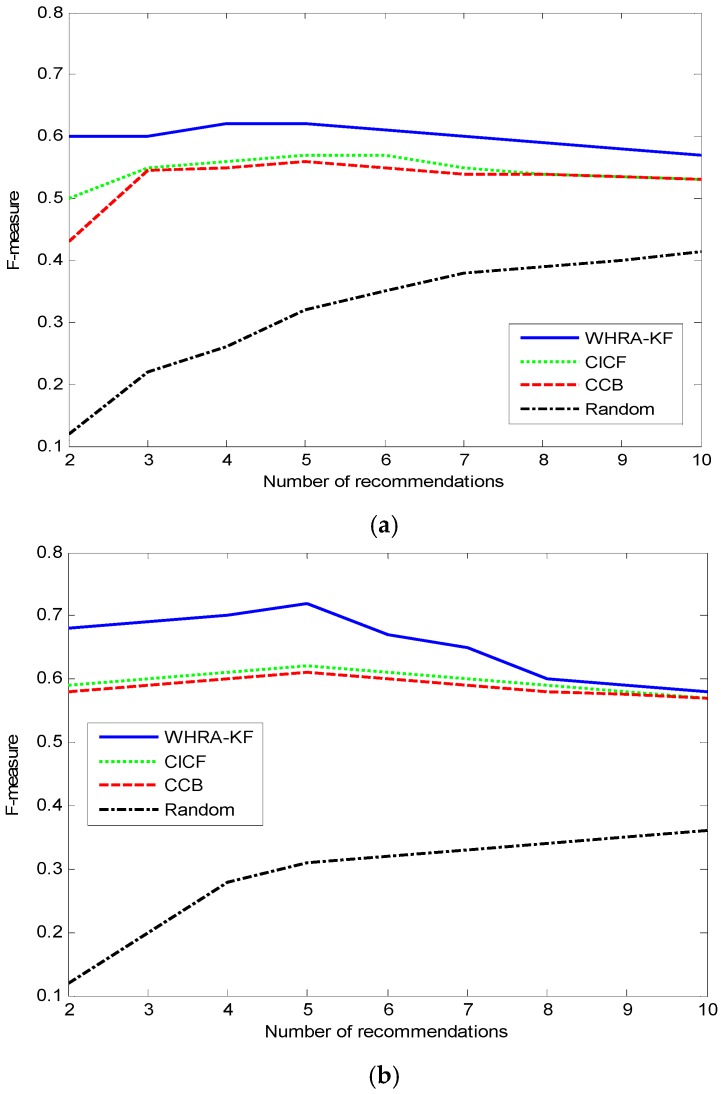
F-measure VS number of recommendation for smart home, (**a**) n=50; (**b**) n=100.

The F-measure value along with the number of recommendations in the two datasets is shown in Figure 10.

For the F-measure values, the value of each algorithm, such as WHRA-KF, CICF and CCB, is small in the end and big in the middle except for the Random recommendation method. The F-measure value of the Random algorithm increases along with the number of recommendations. When *N* is near 5, we get the highest F-measure value for both types of smart home.

**Figure 11:**
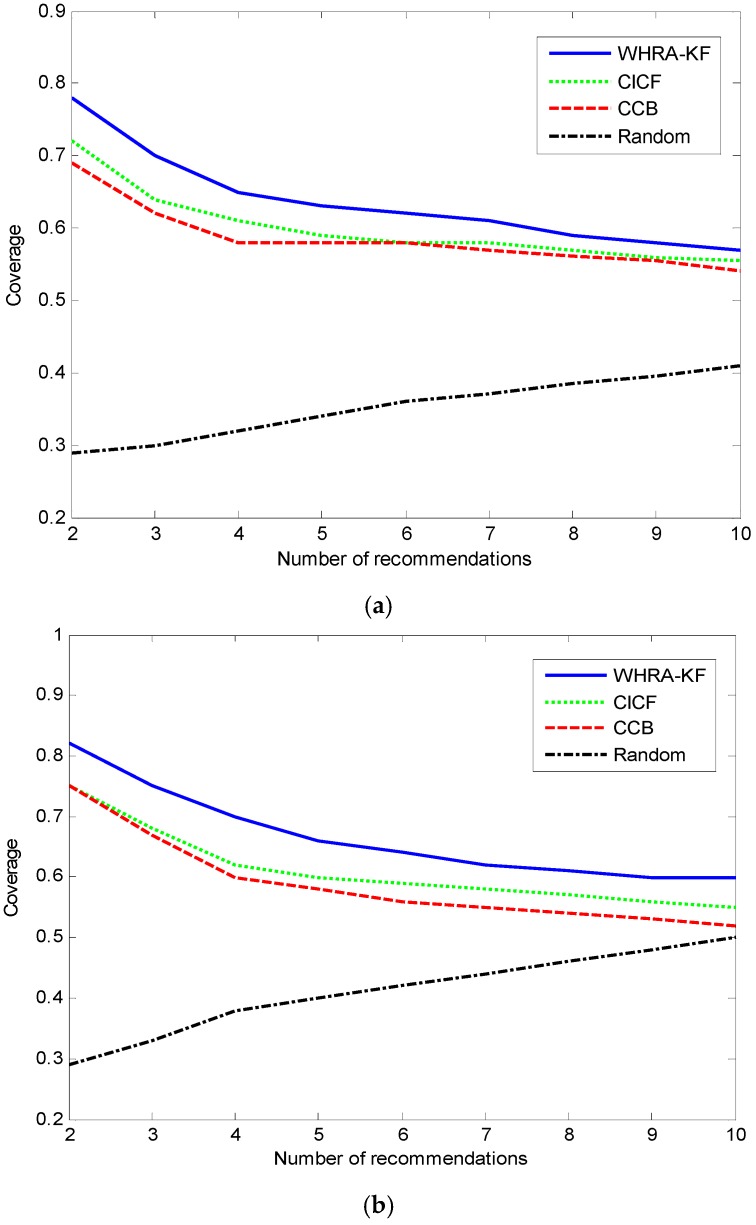
Coverage VS number of recommendation for smart home, (**a**) n=50; (**b**) n=100.

Figure 11 shows the relation between coverage and number of recommendations. Compared to the Random algorithm, WHRA-KF, CICF and CCB have better coverage as the number of recommendations increases. The number of sensors has little influence on the coverage, as seen by comparing the Figure 11a,b. Based on the experimental results above, we can see that the coverage of our approach is higher than that of the other recommendation algorithms, especially when *N* ≤ 10 regardless of the number of sensors. It can also be inferred from the results that the algorithm works better in the deluxe smart home. Actually more sensors in the deluxe smart home than the standard smart home can provide more detailed contextual information about the users and their environment, and the proposed algorithm can effectively make use of this information.

**Figure 12:**
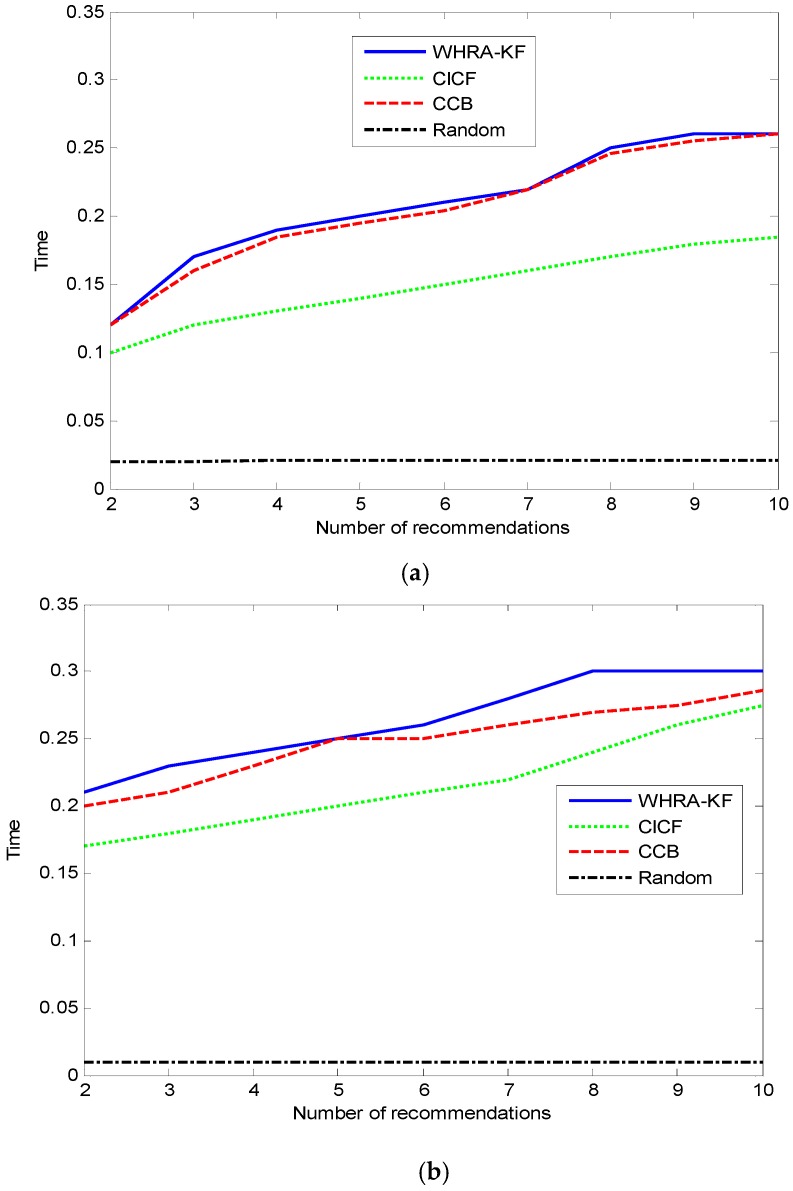
Time VS number of recommendation for smart home, (**a**) Standard smart home (n=50); (**b**) Deluxe smart home (n=100).

The average time cost of generating a recommendation is shown in Figure 12. Clearly, the Random method has the lowest time cost due to its simple idea and theory. Compared to the standard smart home, the deluxe one has a higher time cost when the number of recommendations is the same. That is because more sensors will provide more contextual information, and this results in the recommender systems having to spend more time for processing. Owing to its hybrid characteristics, the time cost in our proposed algorithm is more than CCB and CICF, and there is no large gap among them. This is a benefit of the real-time features of the Kalman-Filter model. It is also proved that our proposed system can be applied in real-time environments. 

It is proved that the proposed hybrid algorithm has better performance, gets more reasonable Recall rate and Precision rate, and has the ability to promote and enhance the quality of recommendations in most environments.

### 5.4. Influences of Sensor Networks

Although previous classical on-demand methods have been proposed to address the problem of power saving, the scheme should be customized to satisfy the differences and diversity of every user in the smart home. As our proposed approach is able to effectively predict the user activities, we can predict when and where the sensors should work in return. We have combined our method with power saving schemes for smart home sensor networks. We compare WHRA-KF and Collaborative Weighted Clustering Algorithm (CWCA) [47] in sensor networks.

**Figure 13:**
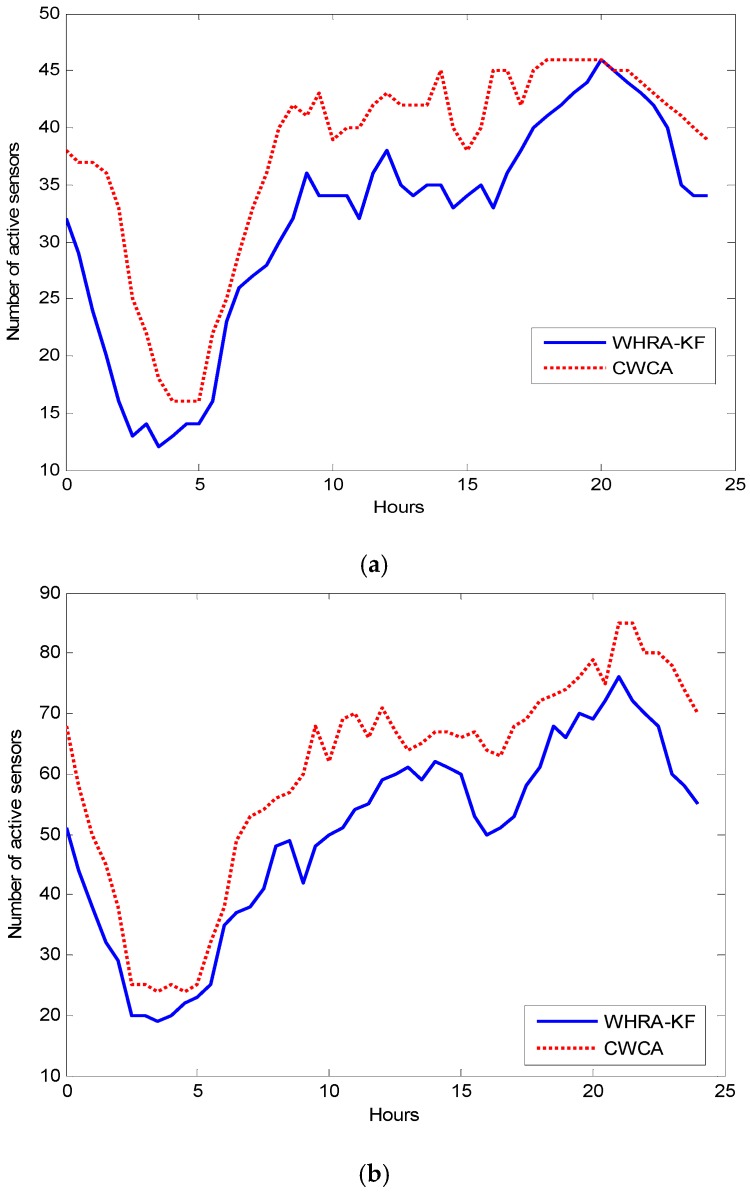
Active sensors over a period of a day on average, (**a**) n=50; (**b**) n=100.

Figure 13 shows the number of active sensors over a period of a day on average for one month time after using WHRA-KF compared with the situation without WHRA-KF. It could be seen that the number of active sensors is obviously smaller for WHRA-KF than the other scenario. Most of the time, the difference between them is more than 15. We can conclude that new scheme can effectively set the sleep times of sensors. 

**Figure 14:**
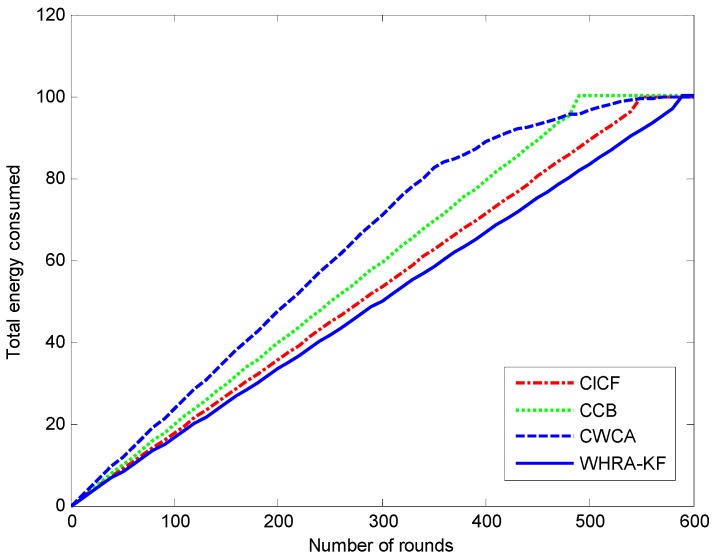
Total energy consumed by the four recommendation algorithms.

Figure 14 shows the energy consumption of the network during the simulation runs for the four recommendation algorithms. It can be observed that the WHRA-KF uses less energy compared to CICF, CCB and CWCA in each round.

**Figure 15:**
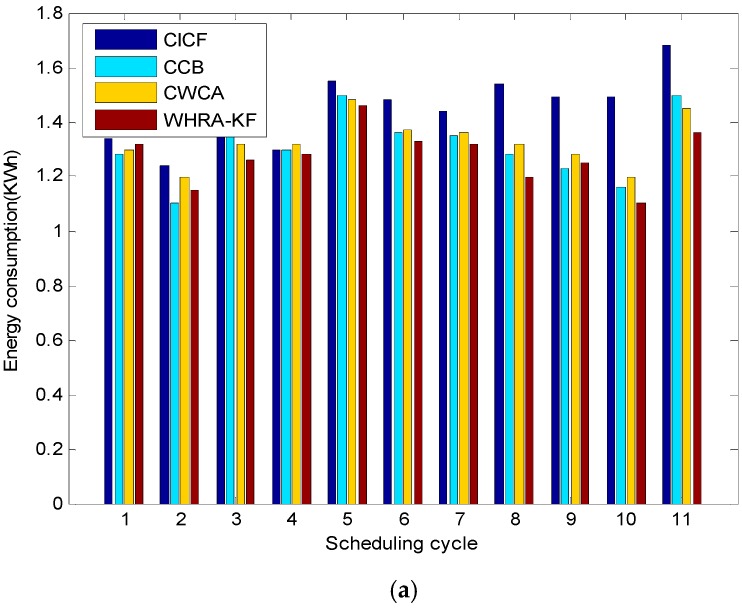

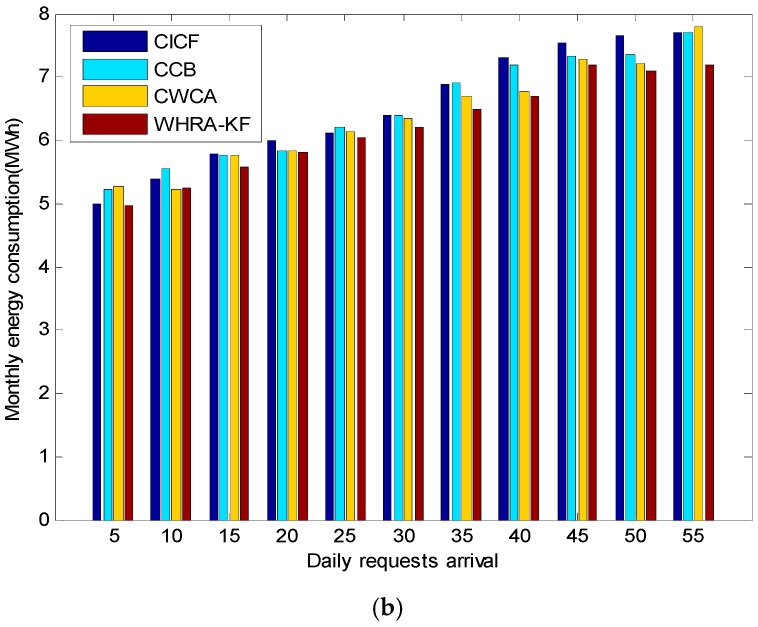
Comparison of the energy consumption for the four recommendation algorithms, (**a**) Different scheduling cycle; (**b**) Different daily requests arrival.

Figure 15 shows the comparison of the energy consumption for different scheduling cycles and daily request arrivals, respectively. It is worth mentioning that the energy consumption plotted in Figure 15a varies depending on the scheduling cycle. It shows that CICF, CWCA and CCB consume on average 1.45, 1.31 and 1.34 kWh per scheduling cycle, respectively. Moreover, the proposed WHRA-KF algorithm achieves a minimum energy consumption of 1.28 kWh per scheduling cycle, with over 20% energy savings compared to the other algorithms. As we can see from Figure 15, WHRA-KF shows more excellent performance in most cases and achieves greater energy savings. It is worth noting that WHRA-KF starts from several initial values simultaneously, and the dependence on the initial values can be effectively reduced so as to speed up the overall search speed.

## 6. Conclusions

With the rapid growth of wireless sensor applications, the user interfaces and configurations of smart homes have become complicated and inflexible so users usually have to spend a lot of time in searching and completing their expected actions. Weighted hybrid recommendation algorithms are a popular research topic in the field of personalized recommendation systems, in which the weight optimization method is key. The specific contributions of this paper include:

(1) A weighted hybrid recommender system based on a Kalman Filter model is proposed for smart home big data analytics for enhanced living environments;
(2) A weighted hybrid recommendation algorithm based on a Kalman Filter is proposed, which can predict and revise the weight of each system component dynamically.

Our experimental results show that the hybrid recommender system can optimize the distribution of weights of each component, and get more reasonable Recall and Precision rates.
